# Facile Fabrication of Amorphous Photonic Structures with Non-Iridescent and Highly-Stable Structural Color on Textile Substrates

**DOI:** 10.3390/ma11122500

**Published:** 2018-12-08

**Authors:** Yichen Li, Liqin Chai, Xiaohui Wang, Lan Zhou, Qinguo Fan, Jianzhong Shao

**Affiliations:** 1 Engineering Research Center for Eco-Dyeing and Finishing of Textiles, Ministry of Education, Zhejiang Sci-Tech University, Hangzhou 310018, China; liyichen131@163.com (Y.L.); chailiqin0101@163.com (L.C.); xhwang0418@123.com (X.W.); lan_zhou330@163.com (L.Z.); 2 Department of Bioengineering, University of Massachusetts Dartmouth, North Dartmouth, North Dartmouth, MA 02747, USA; qfan@umassd.edu

**Keywords:** amorphous photonic structures, structural color, non-iridescent, color stability, textile substrates

## Abstract

Amorphous photonic structures with non-iridescent and highly-stable structural color were fabricated via a simple one-step spray-coating technique. With this strategy, the obtained films on textile substrates presented short-ordered and amorphous photonic structures (APSs) similar to the amorphous nanostructures of avian feathers. The structural color presented the same hue when viewed at different angles and could be well controlled by varying the diameters of the SiO_2_ nanospheres. The prepared fabrics with structural color exhibited high color stability due to stability in both the assembled physical structure and the refractive index. The high stability of the assembled physical structure was attributed to the cementing effect of Poly(methylmethacrylate-butylacrylate) P(MMA-BA) existing between textile substrate and SiO_2_ nanospheres and among SiO_2_ nanospheres, while the high stability in the refractive index was contributed by the liquid-resistance achieved by both the surface roughness and the low-surface-energy of the as-sprayed APSs. With the resistances to external forces and liquid invasion, the non-iridescent brilliant structural color of the as-prepared fabrics could be kept steady. In this study, an approach of fabricating APSs with non-iridescent and stable structural color was established to enhance its potential application in structural coloration of textiles, and other color-related smart textiles.

## 1. Introduction

Structural color, originating from the interaction of visible light with special nanostructures has attracted great attention owing to its non-photofading and eco-friendly properties [1,2,3]. In addition, light interference and/or diffraction from most periodic nanostructures produce iridescent structural colors due to their anisotropy in modulating the light propagation. These iridescent structural colors result in variable and vivid metallic luster effects when they are used as an alternative to pigmentary colors for anti-counterfeiting [4,5], decorations [6], and cosmetics. However, the iridescent effect that changes hue when viewed from different angles often restricts the potential application of structural colors in color-related fields, such as textile colorations, electronic displays, and colorimetric sensors. Hence, fabricating photonic nanostructures with isotropic optical properties has received considerable attention in the functional applications of structural colors.

Interestingly, many living organisms in nature present arresting non-iridescent structural colors due to their intrinsic nanostructures, such as blue bird feathers with an amorphous submicronsized fine air cavities array [7], blue tarantula hairs with rotational symmetry and hierarchy multilayer structures [8], and genus Morpho butterflies with multilayered disorder ridges [9]. Inspired by the non-iridescent structural colors in nature, many artificial nanostructures based on different mechanisms have been fabricated to mimic the intrinsic nanostructures in living organisms. In particular, amorphous photonic structures (APSs), with only short-range order, composed of monodisperse colloidal nanospheres [10,11,12] were the most commonly designed artificial nanostructures owing to their excellent optical properties. In order to obtain the APSs with only short-range order, it is crucial to break down the formation of the long-range order arrangement during the colloidal nanospheres self-assembly process. Therefore, a variety of modified strategies have been proposed, including fabricating in ionic liquid [13,14], fabricating with two different sized particles [10,15,16], fabricating using nanospheres with rough surface [17,18,19,20], fabricating covered with a porous membrane [21], and fabricating with some specific equipment and techniques such as electrophoretic deposition [22,23], inkjet-printing [24], and spray-coating [11,25]. Among these strategies, spray-coating offers unique advantages such as a simple process, lower cost, and rapid and large-scale fabrication, so it is suitable for fabricating APSs on all the surface of all materials.

For the structural color derived from assembled nanostructures, the stability of structural color is of great importance for its application, which depends on the stabilities both in the assembled physical structure and in the refractive index (RI). Usually, the stability in the assembled physical structure is poor due to the weak connecting forces among the colloidal nanospheres [26], so that the prepared APSs could be easily destroyed by some external forces, such as rubbing, bending and washing, resulting in the disappearance of structural colors. On the other hand, the refractive index contrast (RIC) of the prepared APSs may decrease once liquids infiltrate the gaps of nanospheres, resulting in a reduction in saturation of structural color [27,28]. Until now, some novel and effective strategies have been proposed to enhance the stability of APSs with non-iridescent structural color [20,29,30], but due to the restriction of the surface energy of the introduction matter, it may be difficult to maintain the stability of the structural color in some low surface tension solvent.

In this study, non-iridescent structurally colored fabrics with high color stability in both the assembled physical structure and the refractive index were prepared by spraying big-sized organically modified SiO_2_ nanospheres and small-sized P(MMA-BA) copolymer nanoparticles. The stability of the assembled physical structure was strengthened due to the cementing effect of the melted P(MMA-BA) existing between textile substrate and SiO_2_ nanospheres and among SiO_2_ nanospheres. Through the incorporation of alkylated and fluorinated organic component onto the surface of SiO_2_ nanospheres, the prepared structurally colored fabrics presented both superhydrophobic and oleophobic properties. The prepared APSs showed the ability to resist the infiltration of water and oil, so that the RIC of APSs was hardly changed and the structural colors remained stable even as the APSs was immersed into these liquids. It was believed that this facile and low-cost method could provide a novel way for the fabrication of APSs with non-iridescent and highly-stable structural colors on textile substrates and promotes the potential application in eco-coloration of textiles. 

## 2. Materials and Methods 

### 2.1. Materials

Tetraethylorthosilicate (TEOS), ethanol (EtOH), and ammonium hydroxide (NH3·H_2_O) were purchased from Kemiou Reagent Factory (Tianjin, China). Toluene (Tol) and trimethylamine (TEA) were purchased from Gaojing Reagent Factory (Hangzhou, China). Methyl methacrylate (MMA), butyl acrylate (BA), sodium dodecyl benzene sulfonate (SDBS) and potassium persulfate (KPS) were purchased from Aladdin (Shanghai, China). Hexadecyltrimethoxysilane (HDTMS), 1H,1H,2H,2H-Perfluorodecyltrimethoxysilane (FAS) were purchased from Macklin (Shanghai, China). All chemical reagents were used without further purification. Black plain polyester fabric with warp density of 498/10 cm and weft density of 549/10 cm was bought from the local textile market, and the SEM image of the fabric is shown in Figure S1.

### 2.2. Preparation of Organically Modified SiO_2_ Nanospheres

#### 2.2.1. Synthesis of Monodisperse SiO_2_ Nanospheres

The monodisperse SiO_2_ nanospheres were synthesized by the hydrolysis and nucleation reactions with TEOS as the precursor in ethanol solution. In a typical synthetic procedure, 7 mL TEOS, 8 mL H_2_O, and 120 mL ethanol were added to the three-necked round-bottom flask successively with a magnetic agitation (350 rpm) at 25 °C. After 10 min, 3.5 mL NH_3_·H_2_O was introduced into the reactor. The reaction was lasted for 24 h with constant stirring at 25 °C. Finally, the resultant SiO_2_ suspension was thoroughly washed with ethanol three times by centrifugation.

The different diameters of SiO_2_ nanospheres could be obtained by manipulating the concentrations of the reagents (TEOS, NH_3_·H_2_O) used in the synthetic process.

#### 2.2.2. Alkylating of SiO_2_ Nanospheres

**Scheme 1:**
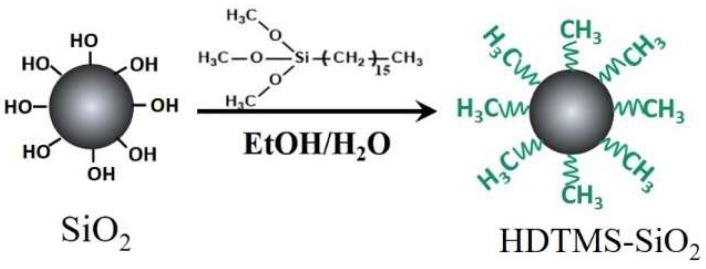
Schematic illustration of the alkylating process of SiO_2_ nanospheres.

Scheme 1 demonstrates the alkylating process of SiO_2_ nanospheres. Firstly, SiO_2_ nanospheres (3 g) were re-dispersed in ethanol (100 mL) with concentration of 3 wt % (Solution A). Then, another mixture (Solution B) including 8 mL water, 2 mL ethanol and a certain amount of HDTMS was slowly added into Solution A with a magnetic agitation (350 rpm) at 75 °C. After 2 h, the alkylating SiO_2_ (HDTMS-SiO_2_) nanospheres suspension was obtained.

#### 2.2.3. Fluorinating of SiO_2_ Nanospheres

**Scheme 2:**
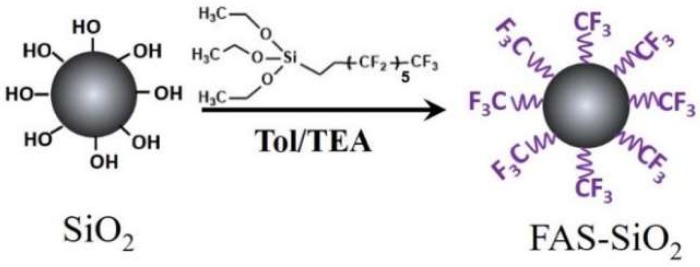
Schematic illustration of the fluorinating process of SiO_2_ nanospheres.

Fluorinating SiO_2_ (FAS-SiO_2_) nanospheres were prepared according to the procedure reported by Xu and Yang [31]. Scheme 2 demonstrates the fluorinating process of SiO_2_ nanospheres. In a typical experiment, 3 g SiO_2_ nanospheres were dispersed in 50 mL toluene, and mixed with 0.5 mL TEA in a 100 mL three-necked round-bottom flask with an intense magnetic agitation at 25 °C. After 5 minutes, 10 mL toluene mixed with certain amount of FAS was slowly added into the three-necked round-bottom flask and reacted for 20 h. Then, FAS-SiO_2_ nanospheres were collected and washed with ethanol three times by centrifugation. Finally, the purified FAS-SiO_2_ were re-dispersed in ethanol with concentration of 3 wt % for further use.

### 2.3. Preparation of P(MMA-BA) Copolymer Nanoparticles

The P(MMA-BA) copolymer nanoparticles were prepared by emulsion polymerization as follows: 90 mL H_2_O, 5 g MMA, 5 g BA and 0.6 g SDS were added to a 250 mL four-necked flask equipped with mechanical stirrer in water bath. After heating the mixer to 80 °C, 0.3 g KPS dissolved in 10 g deionized water was added into the reactor. After 5 h, P(MMA-BA) nanoparticle emulsion with size of about 45 nm were obtained.

### 2.4. Spray-Coating of APSs with Stable Structural Color on Textile Substrates

**Scheme 3:**
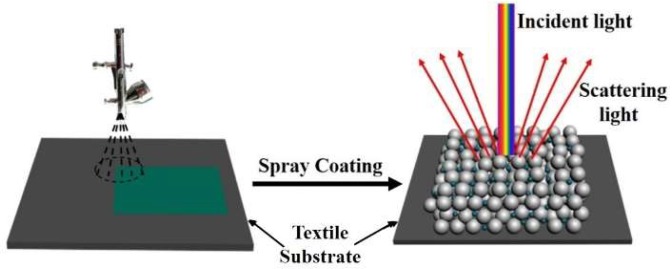
Schematic illustration of the spray-coating process on textile substrates.

The mixed solution for spraying was prepared by re-dispersing modified SiO_2_ nanospheres in ethanol with adding certain amount of 15 mg/mL P(MMA-BA) emulsion, followed by severe ultrasound for 10 min. Plain polyester fabrics were pre-cleaned by a 5 wt % soaping solution in an ultrasonic bath for 15 min, followed by drying at 80 °C at an oven for 2 h before spraying. As shown in Scheme 3, the structurally colored fabrics were fabricated by spraying the mixed solution using an airbrush system with a 0.2 mm nozzle on a hot stage of 60 °C, under a pressure of 50 KPa. The working distance between the nozzle and the fabrics was kept at about 10 cm.

### 2.5. Characterization

The chemical composition of the nanospheres were probed by X-ray photoelectron spectroscopy (XPS; Thermo Fisher Scientific K-Alpha, Waltham, MA, USA,) and Fourier transform infrared spectroscopy (FT-IR, Nicoler 57000, Thermo Scientific, Waltham, MA, USA). Thermogravimetric (TG) analysis was measured with a PerkinElmer instrument (Pyris 1 TGA, Waltham, MA, USA). The average particle sizes of the SiO_2_ nanospheres and P(MMA-BA) particles used in the experiment were measured by a particle zetasize analyzer (Nano-ZS90, Malvern Panalytical, Malvern, UK). The surface morphology of the APSs were observed by a field emission scanning electron microscope (FE-SEM; Ultra 55, Zeiss, Jena, Germany). A thin layer of gold was sputtered on the samples by an ion sputtering instrument (MC1000, Hitach, Tokyo, Japan) for 1 min before observation. The reflection spectra of the structurally colored fabrics were measured by a fiber optic spectrometer (Maya 2000, Ocean Optics, Winter Park, FL, USA) coupled to a six-around-one reflection probe. Digital photos of the structurally colored fabrics were captured with a digital camera (EOS600D, Canon, Tokyo, Japan). Liquid contact angle (CA) and sliding angle (SA) of the structurally colored fabrics were measured by a contact angle goniometer (DSA 20, Krüss, Hamburg, Germany) using liquid droplets of 5 μL in volume. Topographical images were obtained by using atomic force microscopy (AFM; XE-100, Park Systems Corp., Suwon, Korea).

## 3. Results and Discussion

### 3.1. Characterization of the Organically Modified SiO_2_ Nanospheres

**Figure 1:**
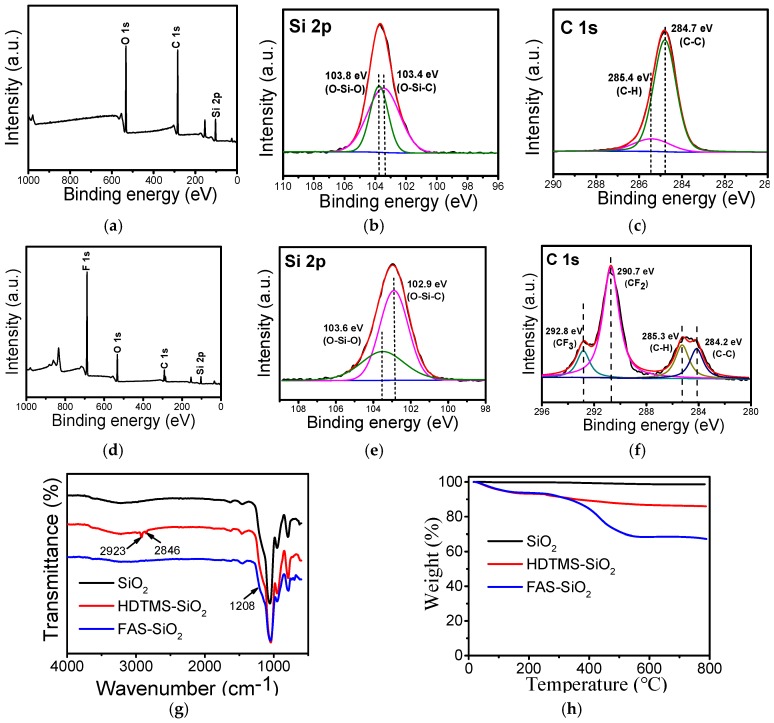
(**a**) Low-resolution, (**b**) Si 2p high-resolution, and (**c**) C 1s high-resolution XPS spectra of HDTMS-SiO_2_ nanospheres; (**d**) Low-resolution, (**e**) Si 2p high-resolution, and (**f**) C 1s high-resolution XPS spectra of FAS-SiO_2_ nanospheres; (**g**) FT-IR spectra of SiO_2_ and modified SiO_2_ nanospheres; (**h**) TGA curves of SiO_2_ and modified SiO_2_ nanospheres.

The organically modified SiO_2_ nanospheres were prepared by the dehydration condensation between the silicon hydroxyl of silane coupler and the hydroxyl of SiO_2_ nanospheres. The chemical composition and surface function groups of the as-prepared HDTMS-SiO_2_ and FAS-SiO_2_ nanospheres were investigated by X-ray photoelectron spectroscopy (XPS), with the full wide-scan spectra confirming the presence of Si, C, and O in HDTMS-SiO_2_ (Figure 1a), as well as Si, C, O, and F in FAS-SiO_2_ (Figure 1d), respectively. High-resolution Si 2p spectra of HDTMS-SiO_2_ (Figure 1b) further deconvoluted into two peaks at 103.4 and 103.8 eV, which were assigned to O–Si–O and O–Si–C moieties, respectively. C 1s spectrurum (Figure 1c) featured signals corresponding to C–C and C–H, respectively. In addition, the high-resolution Si 2p and C 1s spectra of FAS-SiO_2_ in Figure 1e,f confirmed the existence of O–Si–O, O–Si–C, C–C, C–H, CF_2_, and CF_3_ in FAS-SiO_2_ nanospheres. 

To further confirm the organically modification on SiO_2_ nanospheres, the comparison of FT-IR spectra of the as-synthetic SiO_2_ nanospheres and the modified SiO_2_ nanospheres has been performed. As shown in Figure 1g, compared to the as-synthetic SiO_2_, new vibration bands emerged on the curve of HDTMS-SiO_2_ at the positions of 2846 and 2923 cm^−1^, respectively, which were assigned to the –CH_2_– stretching and –CH_3_ stretching of HDTMS. After being modified with FAS, a characteristic peak of 1208 cm^−1^ can be discerned, which was accordant with the stretching of C–F. Notably, the C–F stretching peak was not very obvious due to the overlapping with the stretching peak of Si–O–Si at 1040.6 cm^−1^. As shown in Figure 1h, there were obvious weight losses in the two kinds of modified SiO_2_ nanospheres during the heating process, which could be attributed to the decomposition of organic segments on SiO_2_ nanospheres. Thus, it could be concluded that HDTMS and FAS have been successfully grafted on the surface of SiO_2_ nanospheres. The SEM images in Figure S2a reveals that the as-synthetic SiO_2_ nanospheres were monodispersed in size and spherical in shape. Moreover, after modified with HDTMS and FAS, the modified nanospheres also have highly uniform size and regular spherical morphology (Figure 1b,c), which indicates a potential possibility for the construction of excellent APSs.

### 3.2. Non-Iridescent Structural Color of the as-Prepared Fabrics

In this study, the typical sprayed solution was composed of homogeneously dispersed SiO_2_ nanospheres, P(MMA-BA) copolymer nanoparticles and a low boiling point solvent (ethanol). Under the high air pressure, the mixed suspension quickly flew out from the nozzle to the fabric substrates. Owing to the low boiling point of the solvent, the solvent quickly volatilized during the process from the nozzle to textile substrates. Once SiO_2_ nanospheres reached the fabric substrates, the solvent was completely volatilized and the location of SiO_2_ nanospheres was fixed. Thus, based on the above rapid phase-transition process, a APSs formed on the surface of textile substrate.

**Figure 2:**
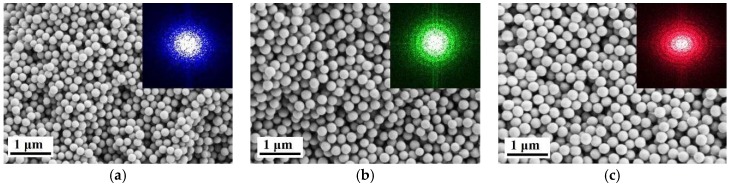
SEM morphology of the APSs, the diameter of HDTMS-SiO_2_ nanospheres were 220 nm (**a**), 270 nm (**b**), and 300 nm (**c**), respectively; inset are the two-dimensional Fourier Transform of the SEM images.

Figure 2 shows the SEM morphology of the as-sprayed APSs on textile substrates fabricated with three different sized HDTMS-SiO_2_ nanospheres of 220 nm, 270 nm, and 300 nm, respectively. It was obvious that these arrays exhibit a typical short-range order but no long-range periodicity. In addition, the inset in Figure 2 shows the corresponding two-dimensional Fourier Transform (2D FTT) of the above SEM images. It can be noted that the 2D FTT pattern displays bright concentric circles, indicating the existence of spatial correlation lengths between the nanospheres that are spatially isotropic and has short-range orders. 

**Figure 3:**
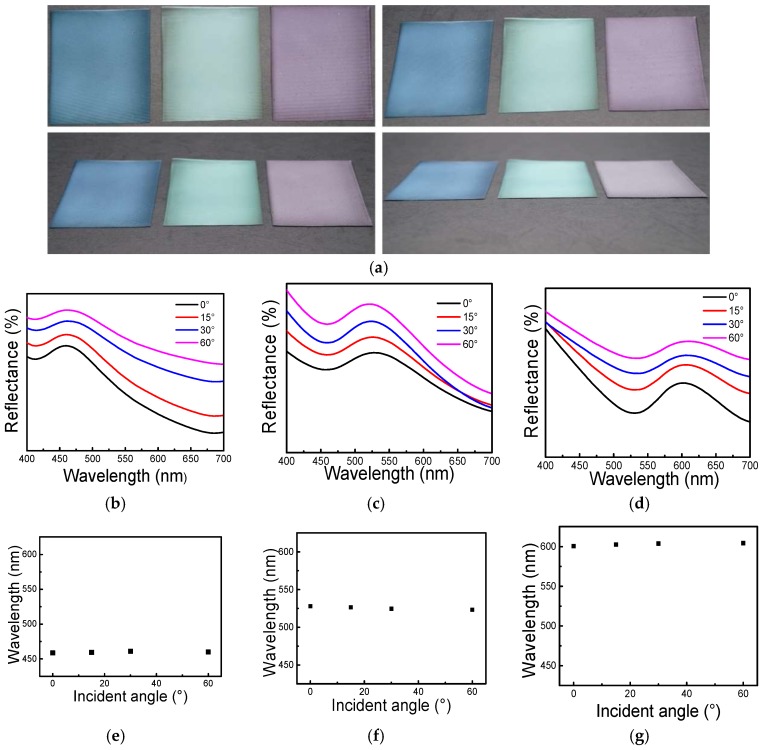
Optical properties of the non-iridescent structurally colored fabrics. (**a**) Photographs of the blue, green and red structurally colored fabrics under different viewing angles. (**b**–**d**) Reflectance spectra of the blue, green and red structurally colored fabrics with different viewing angles. (**e**–**g**) Plots of the positions of the reflection peaks of the three kinds of structurally colored fabrics versus the light incident angle.

**Figure 4:**
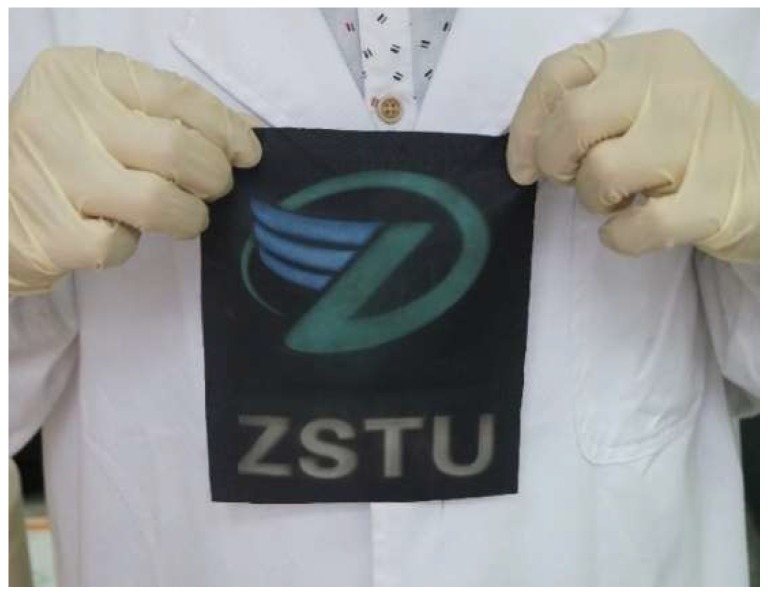
Structural color pattern of our university logo on polyester fabric.

The unique structural features of the APSs contribute special optical properties of the as-sprayed fabrics. Figure 3a presents the images of the as-prepared fabrics under different viewing angles by spray-coating. Clearly, the as-prepared fabrics own wide viewing angles display homogeneous and virtually identical structural colors of blue, green and red at different viewing angles under the D65 light source. To quantify the optical properties of the as-sprayed structurally colored fabrics, UV-vis spectra in reflection mode was applied to measure the reflection spectra under different detection angles (Figure 3b–d). It was noted that the reflection peaks of the blue, green and red fabrics were constant at the wavelength of about 460 nm, 525 nm and 600 nm when detection angles varied from 0° to 60°, respectively. In particular, the reflection peaks under different detection angles remain at approximately the same position, which indicates the non-iridescence of the structurally colored fabrics (Figure 3e–g). Moreover, structural color pattern could be sprayed in large-scale on polyester fabric by the aid of masks (Figure 4).

### 3.3. Stability of the Assembled Physical Structures on Fabric Substrates

The introduction of P(MMA-BA) copolymer particles could significantly enhance the stability of the assembled structures on fabric substrates. In the as-sprayed APSs, the soften P(MMA-BA) copolymer will fill in the interstices of the SiO_2_ nanospheres and act as an adhesive binder to establish a strong connection between the textile substrate and SiO_2_ nanospheres and among the SiO_2_ nanospheres, just like cement binding stones in a sturdy stone wall. 

**Figure 5:**
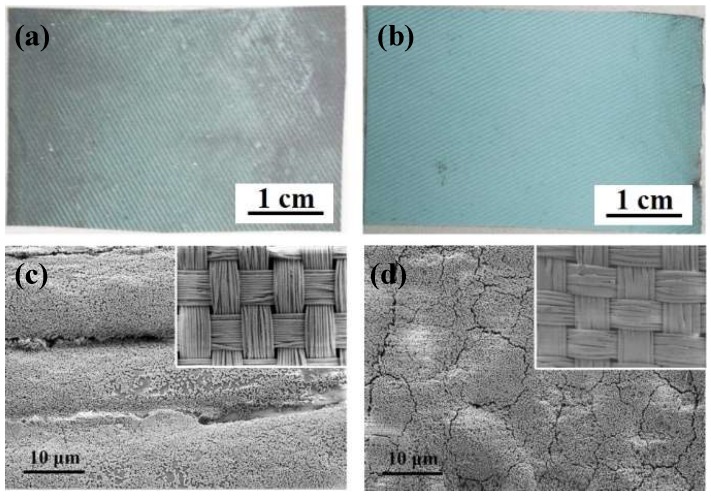
Photograph (**a**,**b**) and SEM images (**c**,**d**) of SiO_2_ (**a**,**c**) and SiO_2_/P(MMA-BA) (**b**,**d**) APSs after soaping fastness test.

The stability of the assembled physical structures on fabrics were evaluated by a soaping fastness test and a rubbing fastness test, which was usually used as the common test methods for color fastness of textiles. Clearly, after the washing of soaping solution for 10 minutes, the structural color of SiO_2_ APSs on the fabric substrate with no P(MMA-BA) added partly disappeared (Figure 5a,c), and even the gaps among the yarns and fibers of the fabric substrate became exposed. In contrast, the SiO_2_/P(MMA-BA) APSs film remained almost intact after the soaping treatment (Figure 5b,d). 

**Figure 6:**
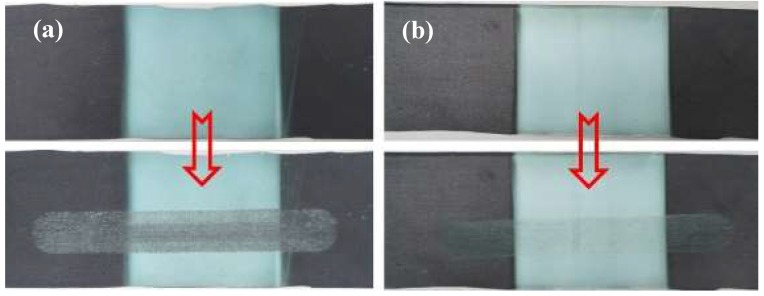
Photograph of SiO_2_ (**a**) and SiO_2_/P(MMA-BA) (**b**) APSs before (up) and after (down) friction fastness test.

Moreover, the results of rubbing fastness test, which was carried out by a rubbing color fastness meter for 10 cycles, were presented in Figure 6. It must be pointed out that after a violent rubbing process, the SiO_2_ APSs film was destroyed; however, for the SiO_2_/P(MMA-BA) APSs, there was less damage in morphology after fraction. Therefore, it was concluded that, compared with SiO_2_ APSs film, the stability of the SiO_2_/P(MMA-BA) APSs was enhanced significantly due to the cementing effect of P(MMA-BA). Clearly, the SiO_2_ nanospheres in SiO_2_/P(MMA-BA) APSs were all locked with each other, verifying the robust mechanical stability of the sprayed APSs (Figure S3).

### 3.4. Stability in the Refractive Index of the APSs

Stability in the refractive index of the APSs is dependent on the wettability of the liquids on APSs. According to current reports [32,33], the combination of surface roughness and low-surface-energy materials is responsible for wettability. Through the alkylated and fluorinated modification, low-surface-energy was successfully endowed for SiO_2_ nanospheres. Meanwhile, surface roughness was obtained by the fast spraying process.

**Figure 7:**
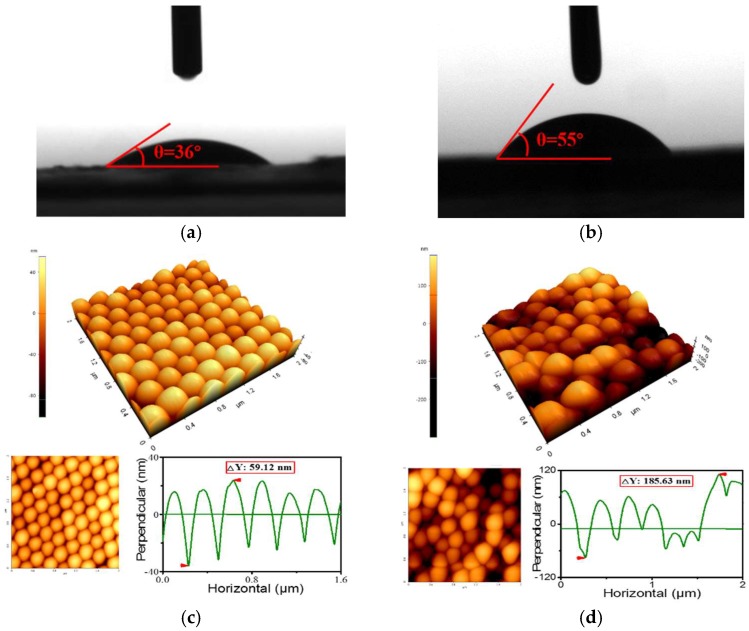
Water CA on SiO_2_ PCs (**a**) and SiO_2_ APSs (**b**); AFM morphology of SiO_2_ PCs (**c**) and SiO_2_ APSs (**d**).

To illustrate the effect of surface roughness of the as-sprayed APSs on wettability, the as-synthetic SiO_2_ nanospheres were used as the basic units to build photonic crystals (PCs) with both short- and long-range order by colloidal self-assembly and APSs with only short-range order by spray-coating on textile substrates. As shown in Figure 7a,b, there was a significant difference in wettability between PCs film and APSs film, with static water contact angles (CA) on the two films of 36° and 55°, respectively. Surface morphology of SiO_2_ PCs film and APSs film was characterized by AFM. It was noted that, compared to PCs film fabricate by colloidal self-assembly, the APSs film exhibited a rougher and disordered surface morphology (Figure 7c,d). It is clear that the vertical distance between the two red labels in Figure 7c,d were 59.12 nm and 185.63 nm, respectively, which revealed the different roughness of the two films. Therefore, it could be confirmed that the increase in surface roughness caused an increase in CA of the SiO_2_ PCs film accordingly.

**Figure 8:**
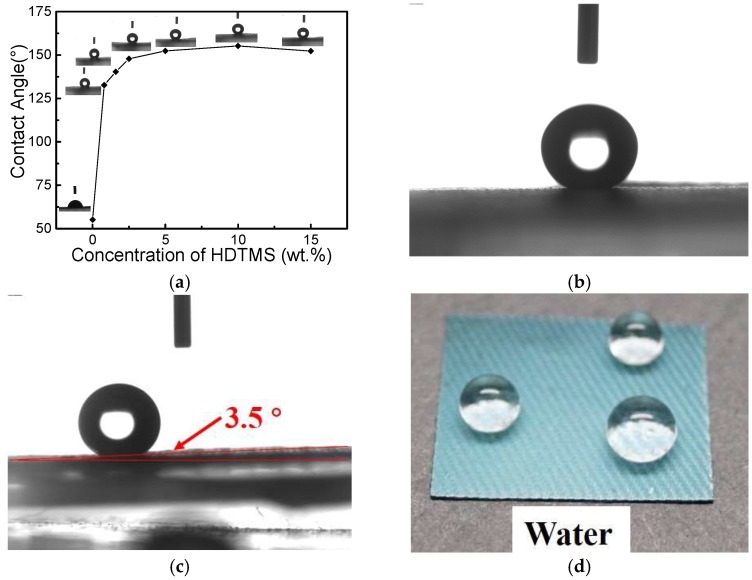
(**a**) The effect of HDTMS concentration (wt %) on water CA of the structurally colored fabrics; (**b**) CA and (**c**) SA of HDTMS-SiO_2_ structurally colored fabric with HDTMS concentration of 10 wt %; (**d**) Three water droplets stand on the structurally colored fabric.

After the modification with HDTMS, the surface of SiO_2_ nanospheres was coated with alkyl chain, resulting in the low-surface-energy of the as-sprayed APSs. From Figure 8a, it was noted that the contact angles (CA) of water on the structurally colored fabrics was significantly increased with as the concentration of HDTMS increased. The CA value in particular reached its maximum when the concentration of HDTMS was 10 wt %. The CA on the green fabric with 10 wt % HDTMS shown in Figure 8b was 155°. Furthermore, the hysteresis of water CA was measured by the sliding angle (SA), and the SA of the structurally colored fabric was 3.5°. Figure 8d shows the digital image of three water droplets standing on the structurally colored fabric. It was clear that the water droplets possess near-spherical shapes, and such a spherical droplet was stably stayed on the fabric surface for longer than 10 min in this study, which indicated that the spray-coated fabric was in a non-wetting state and had superhydrophobic properties.

**Figure 9:**
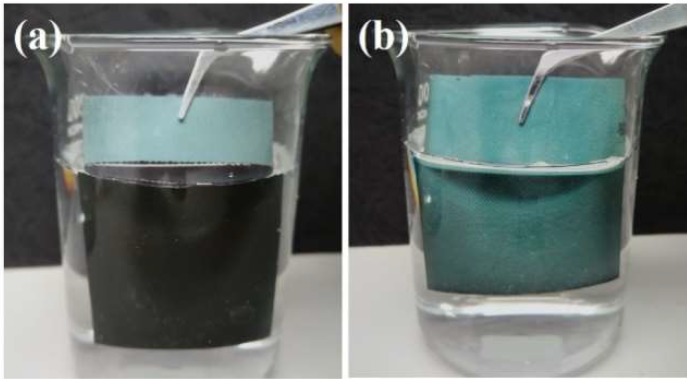
Photograph of SiO_2_ (**a**) and HDTMS-SiO_2_ (**b**) structurally colored fabrics immersed into water.

As shown in Figure 9, it was noted that when the structurally colored fabrics sprayed by as-synthetic SiO_2_ and HDTMS-SiO_2_ were immersed into water, the structural color of SiO_2_ APSs completely disappeared (Figure 9a) because the water could easily penetrate the pores of the SiO_2_ APSs and led to a reduction of RIC of the APSs. Contrarily, due to the low-surface-energy of HDTMS-SiO_2_, the as-sprayed APSs could resist the infiltration of water and kept the RIC unchanged, thus, the structural color of the fabric sprayed by HDTMS-SiO_2_ also exhibited brilliant structural color in water (Figure 9b). In general, through the alkylating of SiO_2_, the sprayed APSs could obtain superhydrophobic properties and present high refractive index stability for water.

**Figure 10:**
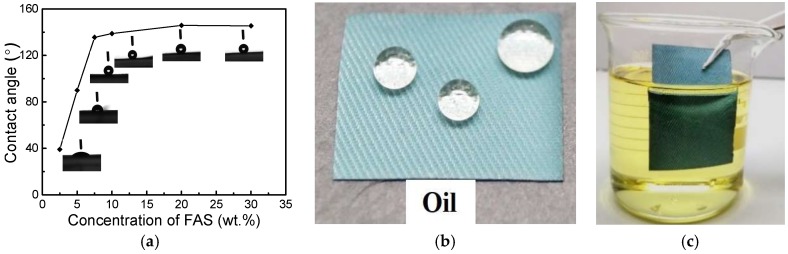
(**a**) The effect of FAS concentration (wt %) on oil CA of the structurally colored fabrics; (**b**) Three salad oil droplets stand on the structurally colored fabric with FAS concentration of 20 wt %; (**c**) Photograph of FAS-SiO_2_ structurally colored fabrics immersed into salad oil.

It is commonly known that oils has a lower surface tension than water, as shown in Figure S4, the surface tensions of water and oil were 71.7 and 34.8 mN/m, respectively. As a result, once the oil droplets were dripped onto the surface of structurally colored fabric sprayed by HDTMS-SiO_2_, they permeated into the APSs immediately, resulting in the disappearance of the structural color (Figure S5). To achieve the purpose of oil-repellent, a lower surface energy was obtained by incorporating fluorinated organic component into SiO_2_ nanospheres to prepare oil-repellent structurally colored fabrics. Figure 10a displayed the effects of the concentration of FAS to the static oil CA of the as-prepared fabrics. In addition, the static oil CA increased with the increasing of the concentration of FAS. The static oil CA reached 146° when the concentration of FAS was 20 wt %, and salad oil droplets can stand on the structurally colored fabric with near-spherical shapes and maintain a non-wetting state for a long time (Figure 10b). Moreover, the structurally colored fabric sprayed by FAS-SiO_2_ nanospheres could maintain its structural color even when infiltrated into salad oil. As a result, through the fluorinated modification of SiO_2_, the sprayed APSs could obtain superoleophobic properties and present high refractive index stability for oil.

## 4. Conclusion

In summary, a novel and effective strategy was proposed for the fabrication of APSs with non-iridescent and highly-stable structural colors on textile substrates by spraying modified SiO_2_ nanospheres and P(MMA-BA) copolymer nanoparticles. Owing to the rapid phase-transition process of the sprayed technique, the formation of the long-range order arrangement was broken and an amorphous photonic structure with only a short-range order which exhibited non-iridescent structural colors was obtained. Moreover, the hue of the structural color was changed with the change of the sizes of the particles. The as-sprayed APSs presented high stability in the assembled physical structures attributed to the cementing effect of the P(MMA-BA) copolymer. Due to the alkylated and fluorinated modification of SiO_2_ nanospheres and the rough surface of the assembled APSs, the prepared fabrics were endowed with superhydrophobic and oleophobic properties with water CA of 155° and oil CA of 146°, respectively, and showed high stability in a refractive index to keep a brilliant structural color when infiltrated into water and oil. It is believed that the facile strategy might open a new method for the application of structural colors in textile coloration, and other color-related smart textiles.

## Supplementary Materials

The following are available online at http://www.mdpi.com/1996-1944/11/12/2500/s1, Figure S1: SEM images of the plain polyester fabric substrate. Figure S2: SEM images of SiO_2_ (a), HDTMS-SiO_2_ (b), and FAS-SiO_2_ (c) nanospheres; Figure S3: Cross-section of SiO_2_ (a) and SiO_2_/P(MMA-BA) (b) APSs; Figure S4: Surface tension of water and salad oil; Figure S5: Salad oil droplet drops on the HDTMS-SiO_2_ structurally colored fabric with HDTMS concentration of 10 wt %.
