# Supplementary material for: Facile Fabrication of Amorphous Photonic Structures with Non-Iridescent and Highly-Stable Structural Color on Textile Substrates
Source: Materials (Basel). 2018 Dec 8;11(12):2500. doi: 10.3390/ma11122500 (PMC6317265; doi:10.3390/ma11122500)
Supplement: Supplementary file 1 [file materials-11-02500-s001.pdf]

Supplementary Materials

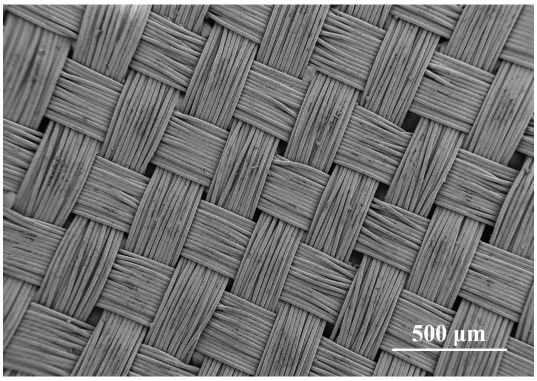

Figure S1 SEM images of the plain polyester fabric substrate.

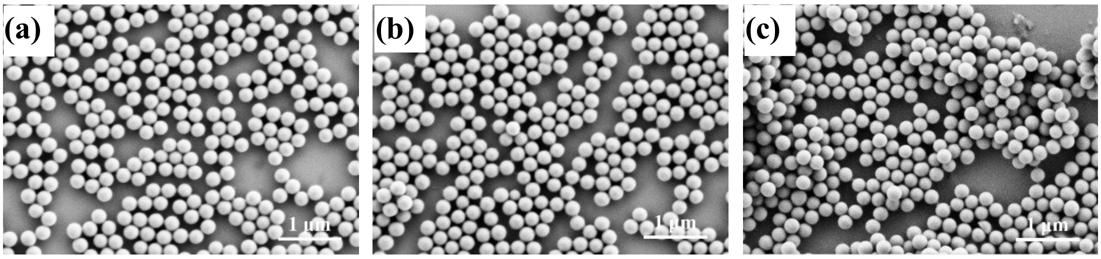

Figure S2 SEM images of SiO_2 (a), HDTMS-SiO_2 (b), and FAS-SiO_2 (c) nanospheres.

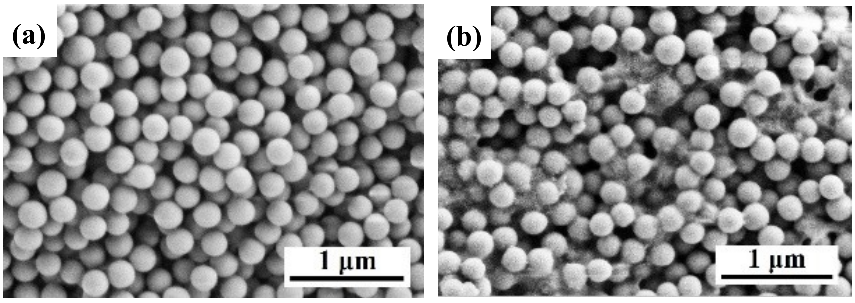

Figure S3 Cross-section of SiO_2 (a) and SiO_2/P(MMA-BA) (b) APSs.

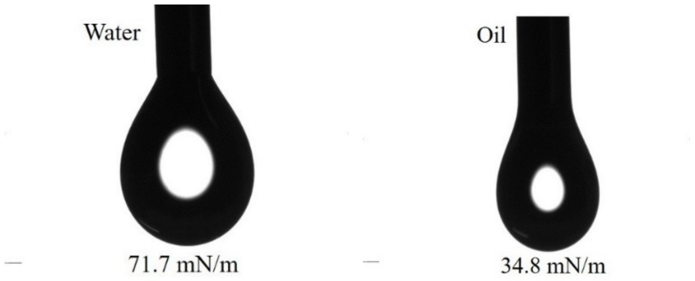

Figure S4 Surface tension of water and salad oil.

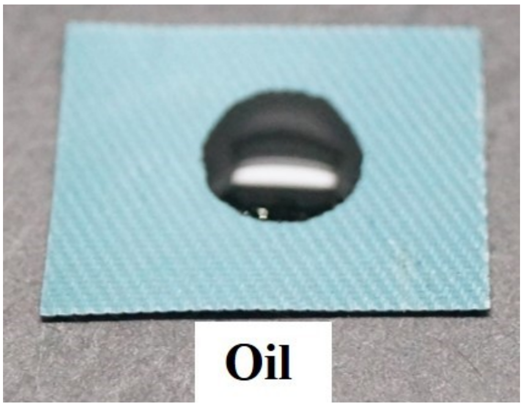

Figure S5 Salad oil droplet drops on the HDTMS-SiO_2 structurally colored fabric with HDTMS concentration of 10 wt%
